# Microbiome markers in HPV-positive and HPV-negative women of reproductive age with ASCUS and SIL determined by V4 region of 16S rRNA gene sequencing

**DOI:** 10.3389/fmicb.2024.1334502

**Published:** 2024-03-14

**Authors:** Anastasiya Peremykina, Valery Cheranev, Andrey Krivoy, Alexander O. Andreev, Zhanna Repinskaia, Aleksandra V. Asaturova, Dmitriy Korostin, Denis Rebrikov, Gyuldana R. Bayramova

**Affiliations:** ^1^Department of Outpatient Clinical Research Development, National Medical Research Center for Obstetrics, Gynecology and Perinatology Named After Academician V.I. Kulakov, Ministry of Health of the Russian Federation, Moscow, Russia; ^2^Center for Precision Genome Editing and Genetic Technologies for Biomedicine, Pirogov Medical University, Moscow, Russia

**Keywords:** squamous intraepithelial lesion, 16S rRNA gene sequencing, HPV, cervicovaginal microbiota, SIL prediction

## Abstract

**Introduction:**

Human papilloma virus (HPV) is the most common sexually transmitted infection worldwide. Cervicovaginal microbiota plays an important role in HPV infection and is associated with the development of squamous intraepithelial lesions (SIL). The natural history of cervical cancer involves reversible changes in the cervical tissue from a normal state, in which no neoplastic changes are detected in the squamous epithelium, to varying states of cellular abnormalities that ultimately lead to cervical cancer. Low-grade SIL (LSIL), like another cytological category - atypical squamous cells of undetermined significance (ASCUS), may progress to high-grade SIL (HSIL) and invasive cervical cancer or may regress to a normal state.

**Methods:**

In this work, we studied cervical canal microbiome in 165 HPV-positive and HPV-negative women of a reproductive age with ASCUS [HPV(+) *n* = 29; HPV(−) *n* = 11], LSIL [HPV(+) *n* = 32; HPV(−) *n* = 25], HSIL [HPV(+) *n* = 46], and the control group with negative for intraepithelial lesion malignancy (NILM) [HPV(−) *n* = 22].

**Results and Discussion:**

HPV16 is the most prevalent HPV type. We have not found any differences between diversity in studied groups, but several genus [like Prevotella (*p*-value = 0.026), Gardnerella (*p*-value = 0.003), Fannyhessea (*p*-value = 0.024)] more often occurred in HSIL group compared by NILM or LSIL regardless of HPV. We have found statistically significant difference in occurrence or proportion of bacterial genus in studied groups. We also identified that increasing of the ratio of *Lactobacillus iners* or age of patient lead to higher chance to HSIL, while increasing of the ratio of *Lactobacillus crispatus* lead to higher chance to LSIL. Patients with a moderate dysbiosis equally often had either of three types of vaginal microbial communities (CST, Community State Type) with the prevalence of *Lactobacillus crispatus* (CST I), *Lactobacillus gasseri* (CST II), and *Lactobacillus iners* (CST III); whereas severe dysbiosis is linked with CST IV involving the microorganisms genera associated with bacterial vaginosis and aerobic vaginitis: *Gardnerella*, *Fannyhessea, Dialister, Sneathia, Anaerococcus, Megasphaera, Prevotella, Finegoldia, Peptoniphilus, Porphyromonas, Parvimonas*, and *Streptococcus*.

## 1 Introduction

Studies on the occurrence and mortality in 36 cancer types conducted in 185 countries show that cervical cancer is the 4th cancer among all cancer types in women (Bray et al., 2018). In developing countries, cervical cancer occurrence and mortality occupies the second place (Qingqing et al., 2021).

The natural history of cervical cancer includes reversible changes in the cervical tissue from a normal state with no neoplastic changes, to varying states of cellular abnormalities in the squamous epithelium that ultimately lead to cervical cancer. The Bethesda “Epithelial cell abnormality: Squamous” category encompasses a spectrum of squamous cell lesions starting from the precancerous lesions of low-grade dysplasia associated with transient human papilloma virus (HPV) infection to higher grade lesions. The two-tiered system of low-grade SIL (LSIL) and high-grade SIL (HSIL) matches the HPV carcinogenic potential. LSIL is now recommended to be used as a diagnostic category to describe HPV transient infection-related changes, while HSIL is used to categorize true precancerous lesion. However, depending on qualitative and quantitative factors, some equivocal morphological features may fall under the category “Atypical Squamous Cells” (ASCs), which are subdivided into two categories; “Atypical Squamous Cells of Undetermined Significance” (ASC-US) or “Atypical Squamous Cells-HSIL cannot be excluded” (ASC-H), based on the suspected underlying lesion LSIL versus HSIL, respectively (Alrajjal et al., 2021).

Development of squamous intraepithelial lesions (SIL) and cervical cancer is mostly caused by the persistence of the human papillomavirus (HPV) (Qingqing et al., 2021; Hu et al., 2022; Zuñiga Martinez et al., 2022). According to the pertinent literature, the group of viruses related to high oncogenic risk includes 13 genotypes (16, 18, 31, 33, 35, 39, 45, 51, 52, 56, 58, 59, 66), two of them (16, 18) being associated with 70% of all cases of cervical cancer (Burd, 2003; Cogliano et al., 2005). The World Health Organization (WHO) estimates that HPV infections worldwide are increasing by 9 to 13% each year (Santella et al., 2022).

The microbial communities of the female reproductive system have been extensively studied over the past decade (Al-Nasiry et al., 2020; Andralojc et al., 2021). According to the published data, one of the risk factors promoting persistent HPV infection is the cervicovaginal microbiota disruption (Ritu et al., 2019; Nieves-Ramírez et al., 2021). Studying the cervicovaginal microbiota in the ‘Human Microbiome’ project revealed that the bacteria colonizing the female genital tract make up 9% of the entire human microbiome (Proctor et al., 2019). In one work, five types of vaginal microbial communities have been outlined (CST – Community State Type) (McClymont et al., 2022; Molina et al., 2022). *Lactobacillus crispatus, Lactobacillus gasseri, Lactobacillus iners*, and *Lactobacillus jensenii* prevail in four CST (I, II, III, and V), respectively, while no *Lactobacillus* species is predominant in CST IV (‘diverse’ group) which is further divided into the following subtypes: CST IV-A comprising the *Anaerococus, Peptoniphilus, Corynebacterium, Prevotella, Finegoldia*, and *Streptococcus* genera; CST IV-В including the *Atopobium, Fannyhessea, Gardnerella, Sneathia, Mobiluncus, Megasphera*genera.

Some authors consider microbial diversity as well as changes in the qualitative and quantitative composition of the cervicovaginal microbiota to be crucial for triggering the proliferation of the stratified squamous epithelial cells and to accompany HPV persistence. Conversely, the viral infection can alter bacterial composition irrespective of the SIL severity looping a ‘vicious circle’ in the development and progression of the disease (Kang et al., 2021; Naidoo et al., 2022; Ntuli et al., 2022). Recently, special attention has been paid to the influence of individual microorganisms on SIL development (Norenhag et al., 2020; Gardella et al., 2022; Lin et al., 2022).

In our study, we aimed to estimate the characteristics of the microbiome by NGS sequencing of 16S ribosomal genes in the HPV-positive [HPV(+)] and HPV-negative [HPV(−)] women of a reproductive age with cervical pathology.

## 2 Methods

### 2.1 Clinical material

From January 2022 to May 2022, 165 women of reproductive age who sought consultation at the Department of Outpatient Clinical Research Development of Kulakov National Medical Research Center for Obstetrics, Gynecology, and Perinatology for diagnosis and treatment of cervical pathology. The patients visited the department because of a wide list of reasons, for example, pregnancy management, absence of menstruation, examination and treatment of female infertility and etc. The primary diagnosis was established based on the results of liquid-based cytology. The cytological material was obtained in accordance with the laws and regulations of the Russian Federation. Air-dried cervical epithelial swabs were prepared by routine Papanicolaou staining. The specimens were collected from patients undergoing cytological and histological examination Departments of Kulakov National Medical Research Center for Obstetrics, Gynecology, and Perinatology (Moscow, Russia). The samples were classified according to the Bethesda system: normal cytology (negative for intraepithelial lesion or malignancy; NILM), low- and high-grade squamous intraepithelial lesions (LSILs and HSILs), or atypical squamous cells of undetermined significance (ASCUS). The final diagnosis was confirmed by histological examination carried out for 76 (46%) women. If the diagnosis had not been verified by histology, patients were placed under dynamic monitoring for cervix condition due to the lack of indications for treatment, and their diagnosis was interpreted by cytological methods. The criteria for inclusion in the study were as follows:

1. The age of women ranging from 19 to 45 years old;
2. The cytological conclusion: ASCUS, low-grade squamous intraepithelial lesion (LSIL), high-grade squamous intraepithelial lesion (HSIL), and negative for intraepithelial lesion or malignancy (NILM);
3. The provided written informed consent for enrollment in the study.

The criteria for exclusion were as follows:

1. Pregnancy;
2. Lactation;
3. Treatment with antibiotics in the previous 14 days;
4. Neuropsychiatric diseases;
5. Acute inflammatory diseases;
6. Renal, liver, or lung dysfunction at the decompensation stage.

This study conformed to the principles of the Declaration of Helsinki. The involved human participants were reviewed and approved by the Local Ethics Committee at the FSBI “National Medical Research Center For Obstetrics, Gynecology And Perinatology Named After Academician V.I.Kulakov” Ministry of Health of the Russian Federation (protocol no. 3 of 26 March 2020).

### 2.2 HPV testing

HPV testing was performed by qPCR using the reagent kit for detecting, genotyping, and identifying of the HPV viral load by PCR “HPVquant-21” (DNA technology, Russia) and involved 21 HPV types (6, 11, 16, 18, 26, 31, 33, 35, 39, 44(55), 45, 51, 52, 53, 56, 58, 59, 66, 68, 73, 82).

### 2.3 Collecting the biomaterial for metagenomic study

The swab was collected into the 1.5 tubes containing 300 microliters of 20 mM Tris–HCl buffer (pH = 7.2).

### 2.4 DNA isolation and library preparation for sequencing

DNA was isolated from the biomaterial using the DNeasy Blood and Tissue kit (Qiagen, United States) following the manufacturer’s instructions. Quality control of the isolated prokaryotic DNA was performed by qPCR using the primers recognizing the V4 DNA region encoding 16S rRNA (515F and 806R) (Parada et al., 2016).

The libraries were prepared for sequencing in two steps. At the first step, the V4 region of 16S rRNA was amplified using the primers to the prokaryotic V4 DNA region encoding 16S rRNA (515F and 806R) containing technical sequences for the MGI adapters. At the second step, amplification was performed using primers containing unique barcodes and primer technical sequences. The concentrations of prepared libraries were measured by Qubit Flex (Life Techonologies, United States) using dsDNA HS Assay Kit (Life Technologies, United States) following the manufacturer’s protocol. The quality of the prepared libraries was assessed using Bioanalyzer 2,100 with the High Sensitivity DNA kit (Agilent Technologies) according to the manufacturer’s instructions. Next, the libraries were circularized and sequenced in the paired-end mode using the DNBSEQG-400 platform with the DNBSEQ-G400RS High-throughput Sequencing Set PE150 kit according to manufacturer’s protocol (MGI Tech). FastQ files were generated using the zebracallV2 software by the manufacturer (MGI Tech).

### 2.5 Sequencing data processing

The obtained overlapping paired reads were merged into unified nucleotide sequences and grouped based on the sequence identity and possible polymerase errors using Qiime2 v.2022.8. Each sequence group was assigned with the taxonomic class (family and genus) using the RDP classificator. Furthermore, each sequence group was aligned using blast v2.13.0 with default settings against the 16S rRNA database followed by determining the species in a read group. Sequence groups with the content of <0.01% in the sample were excluded from the analysis.

### 2.6 Statistical analysis

For statistical analysis, we used compositional data without any transformation to avoid complexity with clinical interpretation. The differences in age between the groups were assessed using One-way ANOVA, followed by multiple pairwise comparisons using the *t*-test with Bonferroni correction. The statistical significance of differences in the ratio of categorical variables (including the qualitative assessment of microorganisms) between the groups was assessed using the Chi-square test, provided that there were no cells in the contingency table with the values below 5. The exact Fisher’s test was used if at least one cell in the contingency table contained a value of <5. Only those microorganisms with the content of >1% in at least two patients were subjected to further qualitative estimation (the frequency of occurrence of a microorganism in the sample). The microorganisms with the content >5% for at least two patients were subjected to further quantitative estimation. The statistical significance of differences upon quantitative assessment between groups was evaluated using the Kruskal–Wallis test. Alpha diversity was assessed using the Shannon and the Simpson index with using amount of reads per taxon. Beta diversity between all groups was assessed by adonis algorithm (Bray-Curtis distance) from “vegan” package (R language). Statistical significance was assessed using the Kruskal–Wallis test for all groups, followed by pairwise comparisons using the Mann–Whitney test with Bonferroni correction. The differences were considered statistically significant at *p* < 0.05. The charts were created using the R programming language (ver 4.2.0). Logistic regression was created using the *‘glm’* and *‘step’* functions from the *‘stats’* package in the R programming language (ver 4.2.0).

## 3 Results

### 3.1 Demographic and clinical indicators did not revealed any significant differences

The study included 165 female participants with the age ranging from 19 to 45 years (mean age: 31 ± 6.3 years). The patients were stratified into 4 groups (Table 1).

**Table 1:** The numbers of patients in groups.

Cytological/Histological conclusion	Total number	Number HPV(+)	Number HPV(−)
NILM (control group)	22	0	22
ASCUS	40	29	11
LSIL	57	32	25
HSIL	46	46	0

**Figure 1:**
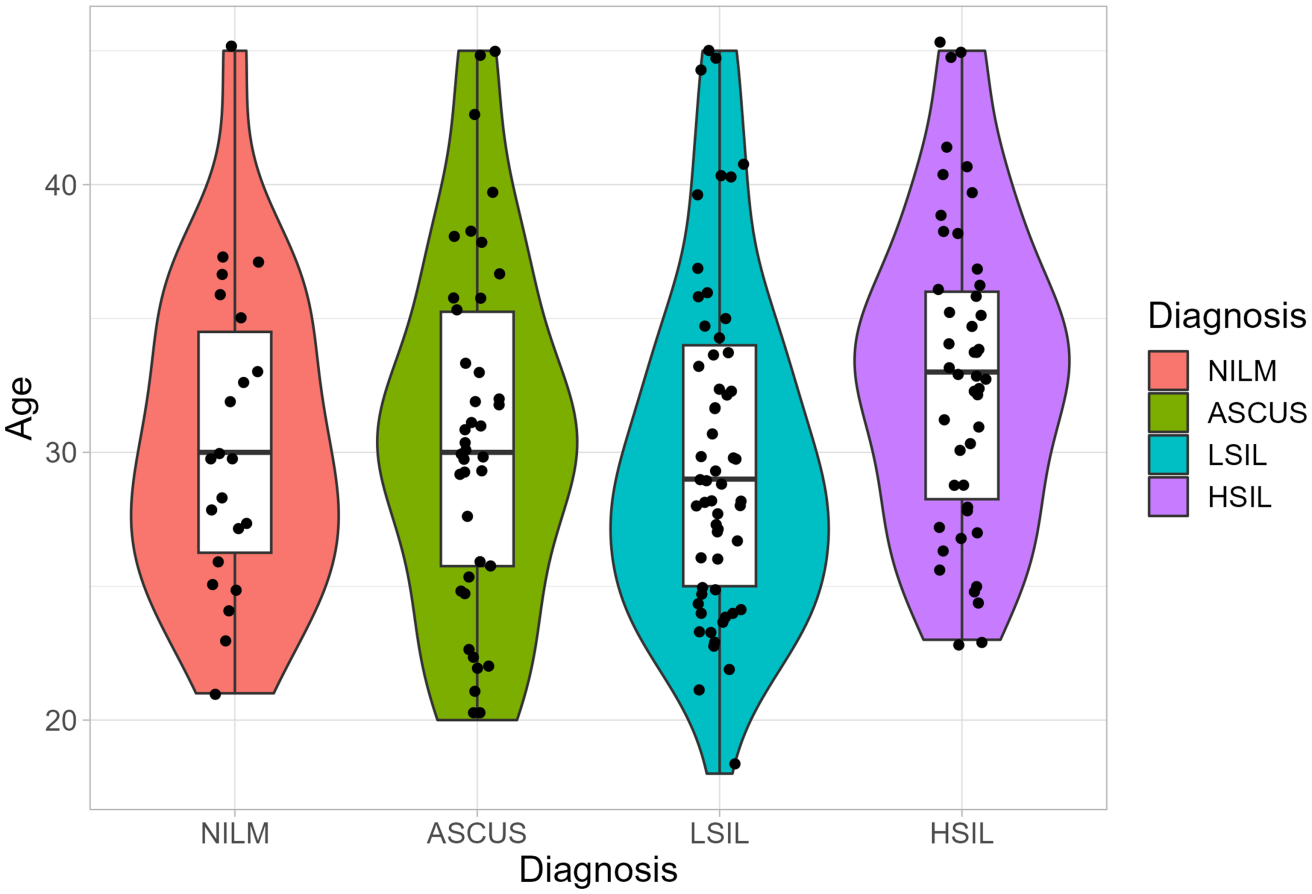
The age distribution in the studied groups. Boxplot shows standard sample metrics (median, Q25–Q75 and Q25 − 1.5 * IQR; Q75 + 1.5 * IQR by whiskers). No statistically significant difference were found between groups.

The mean ages of the patients from the ASCUS, HSIL, LSIL, and NILM groups were 30.7 ± 6.2, 33 ± 5.8, 30 ± 6.3, and 30.4 ± 5.8 years (Figure 1). There were no statistically significant differences between the groups (*p* = 0.093).

**Figure 2:**
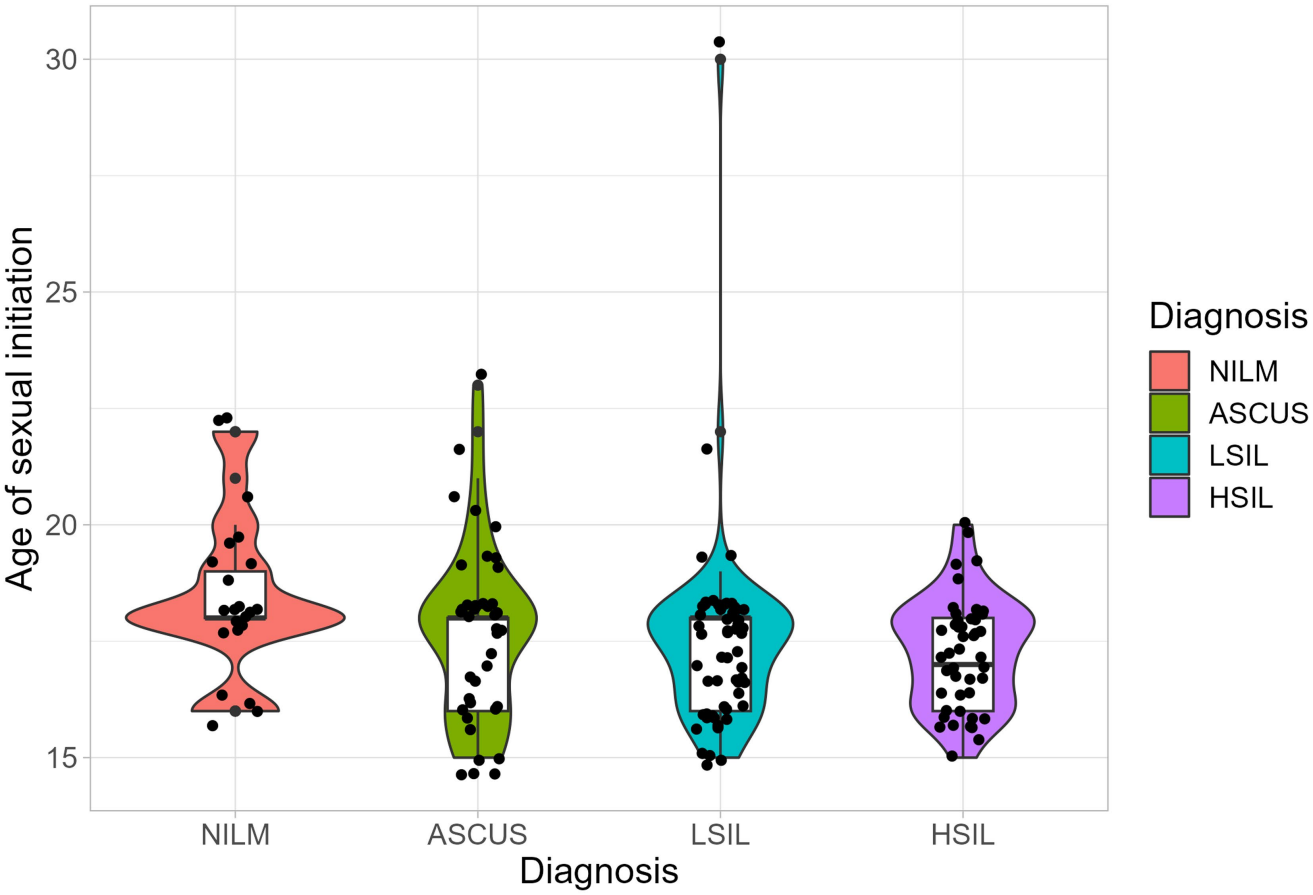
The distribution of the age of sexual initiation. Boxplot shows standard sample metrics (median, Q25−Q75 and Q25 − 1.5 * IQR; Q75 + 1.5 * IQR by whiskers). No statistically significant difference were found between groups.

The median ages of sexual initiation in the ASCUS, LSIL, HSIL, and NILM groups were 18 years (IQR (Q25–Q75) is 16–18 years), 18 years (16–18 years), 17 years (16–18 years), and 18 years (18–19 years), respectively (Figure 2). We detected statistically significant differences in the age of sexual initiation between the LSIL and NILM groups (adjusted *p*-value = 0.018) and between the HSIL and NILM groups (adjusted *p*-value = 0.03). On average, patients from the HSIL and LSIL groups have a lower age of sexual initiation (1 year less) compared to the control group.

Statistical analysis of the patients based on anamnestic and clinical parameters (somatic morbidity, pregnancies, childbirths, contraception methods, surgical interventions, gynecological diseases, and past infectious diseases) did not reveal any significant differences between the groups (the data are shown in Supplementary Table S1).

### 3.2 HPV16 type and overall amount of infected patient was higher in HSIL group

**Figure 3:**
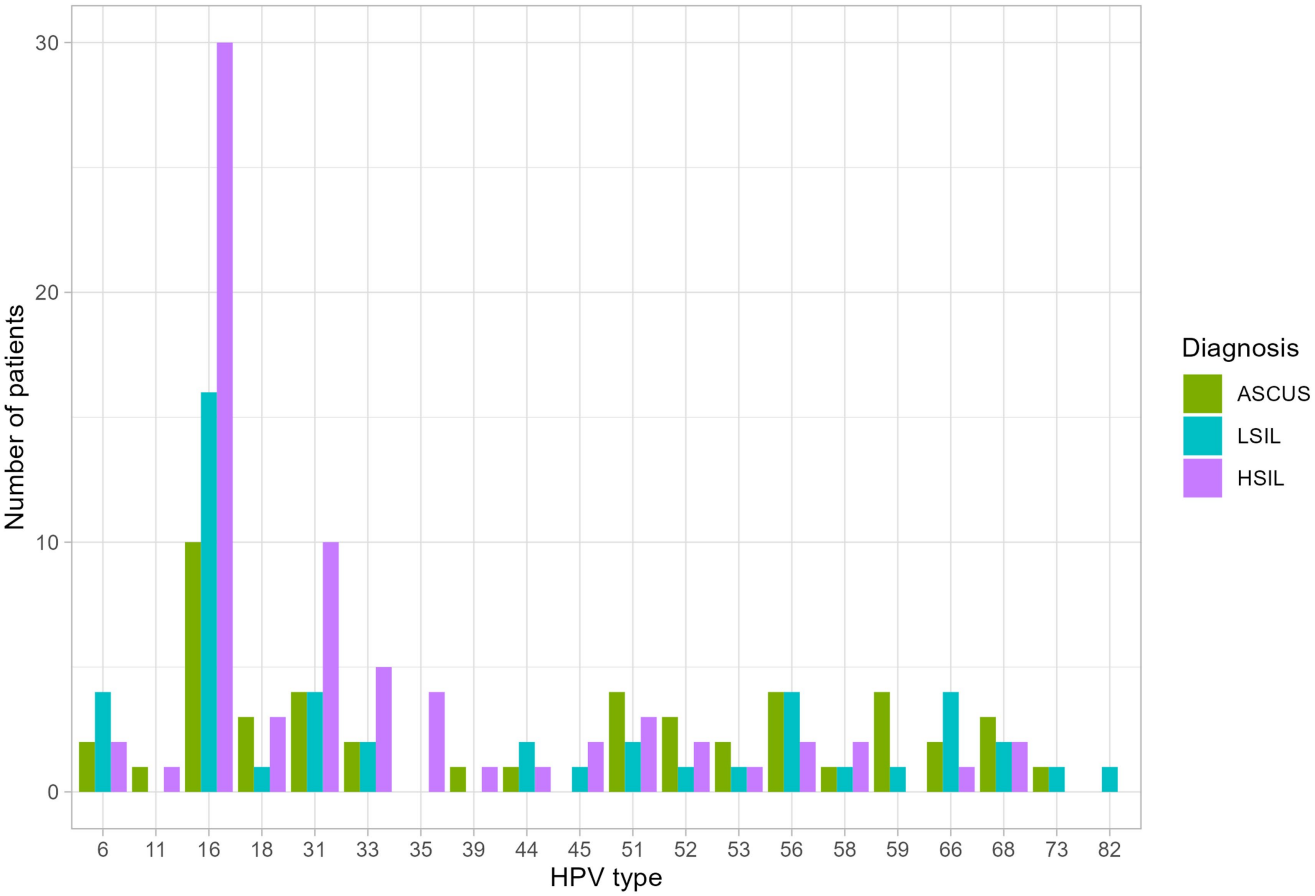
The distribution of HPV types among the studied groups. Statistically significant difference were identified for HPV 16 type in HSIL group and at least one HPV type was higher in the HSIL group.

**Figure 4:**
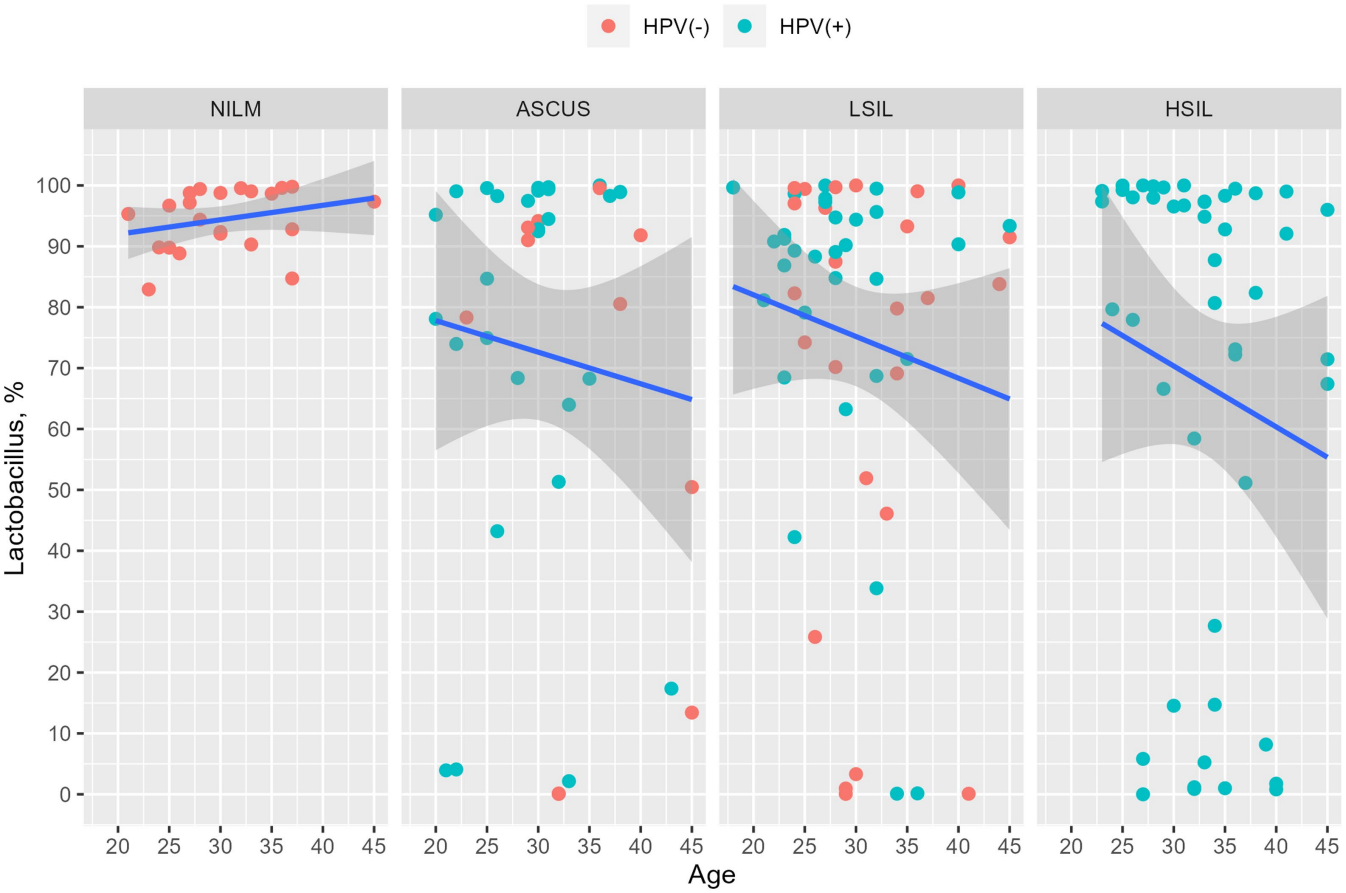
Dependency of the *Lactobacillus* genus representation from age. The HPV-negative and HPV-positive patients are indicated in red and blue, respectively. It was observed that the percentage of *Lactobacillus* is decreased in all groups except NILM. The changes were not statistically significant.

The number of patients with at least one HPV type was higher in the HSIL group (100%) than in the LSIL (72.5%) and ASCUS (56.1%) groups (adjusted *p*-value = 0.0086). The most common HPV type in the LSIL, HSIL, NILM was HPV16. After applying the correction for multiple comparisons, only HPV16 exhibited statistically significant differences (adjusted *p*-value = 0.013) (Tables 2, 3; Figure 3). The Spearman’s correlation coefficient between the age and the Lactobacillus representation across all studied groups was 0.095 being not statistically significant (*p*-value = 0.22), similarly to the correlation coefficient between the age and HPV carriage (rho = −0.059, *p*-value = 0.46). Estimating the correlation between the Lactobacillus representation among the studied groups did not provide any statistically significant results (Table 3). The groups under scrutiny showed the reverse correlation between the age and Lactobacillus representation in the sample. In particular, the correlation coefficient raised from -0.28 in the HSIL group to 0.38 in the control NILM group (Figure 4, Table 3). Estimating the linear correlation between the HPV carriage and age in the HSIL and NILM groups was impossible since they comprised only one HPV carriage group. The ASCUS group showed the negative statistically significant correlation (*p*-value = 0.047) between the age and HPV carriage (Table 3).

**Table 2:** The ratio of HPV types across the groups.

HPV type	ASCUS (*n* = 40)	LSIL (*n* = 57)	HSIL (*n* = 46)
11	1	0	1
**16**	**10**	**16**	**30**
18	3	1	3
31	4	4	10
33	2	2	5
35	0	0	4
39	1	0	1
44	1	2	1
45	0	1	2
51	4	2	3
52	3	1	2
53	2	1	1
56	4	4	2
58	1	1	2
59	4	1	0
6	2	4	2
66	2	4	1
68	3	2	2
73	1	1	0
82	0	1	0
At least one type	**29**	**32**	**46**

Statistically significant differences (adjusted *p*-value < 0.05) in the ratio of HPV types across the groups are highlighted in bold.

**Table 3:** The Spearman’s correlation coefficient and statistical significance between the age and *Lactobacillus* representation.

	The age and the *Lactobacillus* percentage	The age and HPV carriage
Group	*r* (*p*-value)	*r* (*p*-value)
NILM	0.38 (0.078)	–
ASCUS	−0.017 (0.92)	−0.32 (**0.047**)
LSIL	−0.14 (0.29)	−0.23 (0.08)
HSIL	−0.28 (0.059)	–

Rho, the Spearman’s correlation coefficient. Statistically significant correlation (r) in bold.

### 3.3 Evaluation of the alpha and beta microorganism diversity

Statistically significant differences in the alpha diversity between the groups were not detected (Table 4). The control group (NILM) displayed the lowest diversity level. Analyzing all patients as well as only HPV(+) patients revealed the greatest level of diversity in the HSIL group. In the HPV(−) patients, the highest diversity level was observed for the ASCUS group.

**Table 4:** Estimation of the microorganism diversity in the analyzed groups.

Diversity index	NILM	ASCUS	LSIL	HSIL	*p*-value
Shannon	0.41 (0.26–0.54)	0.582 (0.24–1.11)	0.56 (0.33–1.04)	0.81 (0.25–1.25)	0.101
Simpson	0.15 (0.09–0.27)	0.31 (0.11–0.58)	0.28 (0.16–0.52)	0.41 (0.11–0.62)	0.115
		HPV(−)			
Shannon	0.41 (0.26–0.54)	0.82 (0.37–1.54)	0.68 (0.27–1.21)	–	0.064
Simpson	0.15 (0.09–0.27)	0.35 (0.14–0.69)	0.30 (0.11–0.58)	–	0.093
		HPV(+)			
Shannon	0.41 (0.26–0.54)	0.57 (0.2–1.04)	0.52 (0.37–1.00)	0.81 (0.25–1.25)	0.104
Simpson	0.15 (0.09–0.27)	0.27 (0.07–0.53)	0.27 (0.17–0.49)	0.41 (0.11–0.62)	0.108

Data are shown as a median with the interquartile range (Q25–Q75).

The Beta diversity analysis did not revealed any statistically significant difference between groups (*p*-value = 0.15). Pairwise comparison taking into account HPV status also did not show difference (Supplementary Table S8). Pairwise comparison without HPV revealed only one statistically significant difference (*p*-value = 0.036) between HSIL and LSIL group, but after applying multiple comparisons correction it was gone (*p*-value = 0.22).

### 3.4 The qualitative and quantitative estimations of the *Lactobacillus* species did not reveal any significant difference

The qualitative and quantitative estimations of the *Lactobacillus* content in the HPV(+) or HPV(−) patients, and the control group are shown in Tables 5, 6. There was no statistically significant difference in the *Lactobacillus* content between the groups (*p* > 0.05). Below is the list of the species sorted based on their content: *Lactobacillus crispatus, L. iners, L. jensenii, L. gasseri, L. delbrueckii*. It is worth noting that among the *Lactobacillaceae* family, we also detected the species of *Limosilactobacillus* genus (including *L. antri*, *L. coleohominis*, *L. reuteri*, *L. mucosae*) in the HPV(+) patients with ASCUS (2 cases, 5%), LSIL (4 cases, 12.5%), HSIL (3 cases, 6.52%), and in the control group (2 cases, 9.09%). No statistically significant difference was observed between studied groups by genus.

**Table 5:** The qualitative and quantitative characteristics of the *Lactobacillus* species content in the HPV(+) patients and control group.

*Lactobacillus* species	Data	NILM (*n* = 22)	ASCUS HPV(+) (*n* = 29)	LSIL HPV(+) (*n* = 32)	HSIL HPV(+) (*n* = 46)	*p*-value
*L. crispatus*	*n* (%)	16 (72.7)	20 (69)	26 (81.2)	33 (71.7)	0.71
	Median (Q25–Q75)	0.36% (0.01–87.1)	7.04% (0–92)	26.5% (0.2–88.4)	0.56% (0–27.7)	0.28
*L. jensenii*	*n* (%)	9 (41)	12 (41.4)	19 (59.4)	21 (45.7)	0.44
	Median (Q25–Q75)	0% (0–5.3)	0% (0–2)	0.54% (0–5.4)	0% (0–4.1)	0.67
*L. gasseri*	*n* (%)	4 (18.2)	6 (20.7)	9 (28.1)	11 (23.9)	0.85
	Median (Q25–Q75)	0% (0–0)	0% (0–0)	0% (0–0)	0% (0–0.13)	0.90
*L. iners*	*n* (%)	15 (68.2)	14 (48.3)	20 (62.5)	25 (54.4)	0.47
	Median (Q25–Q75)	18.51% (0–82.8)	0% (0–39.4)	0.25% (0–45.4)	0.29% (0–72.2)	0.34
*L. delbrueckii*	*n* (%)	1 (4.5)	–	–	1 (2.2)	0.97
	Median (Q25–Q75)	4% (0–0)	–	–	0.01% (0–0)	0.97

Data are presented as a number of patients in a group that have a certain *Lactobacillus* species (the percentage from the total number of patients per group is given in the parentheses). *p*-value, (Pearson’s Chi-squared test or Exact Fisher’s test). The quantitative metrics are presented as a median with an interquartile range. *p*-value, Kruskal–Wallis test.

**Table 6:** The qualitative and quantitative characteristics of the content of various *Lactobacillus* species in the HPV(−) patients and the control group.

*Lactobacillus* species	Data	NILM (*n* = 22)	ASCUS HPV(−) (*n* = 11)	LSIL HPV(−) (*n* = 25)	*p*-value
*L. crispatus*	*n* (%)	16 (72.7)	7 (63.6)	17 (68)	0.87
	Median (Q25–Q75)	0.36% (0.01–87.1)	0.31% (0–3.2)	7.99% (0–87.5)	0.51
*L. jensenii*	*n* (%)	9 (41)	3 (27.3)	11 (44)	0.72
	Median (Q25–Q75)	0% (0–5.3)	0% (0–0.01)	0% (0–3)	0.51
*L. gasseri*	*n* (%)	4 (18.2)	4 (36.4)	7 (28)	0.49
	Median (Q25–Q75)	0% (0–0)	0% (0–4.3)	0% (0–0.04)	0.54
*L. iners*	*n* (%)	15 (68.2)	8 (72.7)	12 (48)	0.28
	Median (Q25–Q75)	18.5% (0–82.8)	11% (0.03–83)	0% (0–10.5)	0.07
*L. delbrueckii*	*n* (%)	1 (4.5)	–	1 (4)	0.98
	Median (Q25–Q75)	0% (0–0)	–	0% (0–0)	0.97

Data are presented as a number of patients in a group that have a certain *Lactobacillus* species (the percentage from the total number of patients per group is given in the parentheses). *p*-value, (Pearson’s Chi-squared test or Exact Fisher’s test). The quantitative metrics are presented as a median with an interquartile range. *p*-value, Kruskal–Wallis test.

### 3.5 The qualitative and quantitative estimations of the content of microorganisms (except *Lactobacillus* species) in the HPV(+) and HPV(−) patients

In this work, the cervical canal of the women of reproductive age comprised more than 100 various bacterial genera, 44 being the most common (not <1% of reads in two or more samples). Estimating the frequencies of detecting taxons across all the groups studied revealed statistically significant differences in the occurrence frequencies of *Fannyhessea* and *Gardnerella* among the ASCUS, HSIL, LSIL, and NILM groups (Table 7). Pairwise group comparisons showed the differences in the occurrence frequencies between the control group and the groups under scrutiny (ASCUS, HSIL, LSIL). For the *Prevotella* genus, significant differences were found only between the HSIL and NILM groups. In all detected statistically significant differences, these microorganism genera were more common in the groups under investigation (ASCUS, HSIL, LSIL) compared to the NILM group. Other comparisons did not provide any statistically significant results (Supplementary Table S2).

**Table 7:** The qualitative estimation of the microorganism occurrence frequencies in the HPV(+) patients.

Genera	NILM (*n* = 22)	ASCUS (*n* = 29)	LSIL (*n* = 32)	HSIL (*n* = 46)	Difference between all groups	ASCUS versus NILM	LSIL versus NILM	HSIL versus NILM
					*p*-value	*p*-value	*p*-value	*p*-value
Fannyhessea	0 (0%)	5 (17.2%)	2 (6.3%)	10 (21.7%)	**0.036**	**0.062**	0.508	**0.024**
Prevotella	0 (0%)	4 (13.8%)	2 (6.6%)	9 (19.6%)	0.0764	0.12	0.508	**0.026**
Gardnerella	1 (4.6%)	9 (31%)	12 (37.5%)	18 (39.1%)	**0.0132**	**0.03**	**0.008**	**0.003**

*p*-value, (Pearson’s Chi-squared test or Exact Fisher’s test). Statistically significant differences are highlighted in bold.

The qualitative estimation of all groups of HPV(−) patients revealed statistically significant differences for 4 microorganism genera: *Prevotella*, *Gardnerella*, *Porphyromonas*, *Dialister* (Table 8). Pairwise comparisons demonstrated the significant differences only between the ASUS and control groups for all 4 genera. They occurred significantly more frequently in the ASCUS group compared to the control group. Other comparisons did not provide any statistically significant results (Supplementary Table S3).

**Table 8:** The qualitative estimation of the microorganism occurrence frequencies in the HPV(−) patients.

Genera	NILM (*n* = 22)	ASCUS (*n* = 11)	LSIL (*n* = 25)	Difference between all groups	ASCUS versus NILM	ASCUS versus LSIL	LSIL versus NILM
				*p*-value	*p*-value	*p*-value	*p*-value
Prevotella	0 (0%)	3 (27.27%)	4 (16%)	**0.046**	**0.03**	0.65	0.11
Gardnerella	1 (4.55%)	6 (54.55%)	6 (24%)	**0.005**	**0.003**	0.16	0.10
Porphyromonas	0 (0%)	3 (27.27%)	1 (4%)	**0.019**	**0.03**	0.07	1
Dialister	0 (0%)	3 (27.27%)	4 (16%)	**0.046**	**0.03**	0.65	0.11

*p*-value, (Pearson’s Chi-squared test or Exact Fisher’s test). Statistically significant differences are highlighted in bold.

The quantitative estimation of the microorganism content across all studied groups revealed statistically significant differences in case of the *Ureaplasma* genus (Table 9). On average, *Ureaplasma* is more common in the LSIL group than in the other groups. Pairwise comparisons confirmed the statistically significant differences of *Ureaplasma* occurrence between the HSIL and LSIL as well as LSIL and NILM groups. Furthermore, the *Parvimonas* species was shown to occur more frequently in the HSIL group compared to the LSIL group. Significant differences were observed also for the *Parvimonas*, *Streptococcus*, *Staphylococcus*, *Porphyromonas*, *Gardnerella* genera. On average, the representation percentages of *Streptococcus*, *Staphylococcus*, *Porphyromonas*, *Gardnerella* genera were higher in the ASCUS, HSIL, and LSIL groups compared to the NILM group. Other comparisons did not provide any statistically significant results (Supplementary Table S4).

**Table 9:** The quantitative estimation of the microorganism representation in the HPV(+) patients.

Genera	NILM (*n* = 22)	ASCUS (*n* = 29)	LSIL (*n* = 32)	HSIL (*n* = 46)	Difference between all groups	ASCUS versus NILM	HSIL versus LSIL	HSIL versus NILM	LSIL versus NILM
					*p*-value				
Gardnerella	0 (0–0.6)	0 (0–19.2)	0.05 (0–14)	0 (0–23)	0.27	0.15	0.91	0.11	**0.04**
Porphyromonas	0 (0–0)	0 (0–0.01)	0 (0–0)	0 (0–0.5)	0.20	0.12	0.33	**0.04**	0.14
Staphylococcus	0.05 (0–0.2)	0.02 (0–0.2)	0 (0–0.2)	0 (0–0.2)	0.22	0.44	0.81	**0.043**	0.10
Streptococcus	0 (0–0.3)	0.06 (0–6.3)	0 (0–2.9)	0 (0–1.2)	0.07	**0.012**	0.87	0.20	0.311
Parvimonas	0 (0–0)	0 (0–0)	0 (0–0)	0 (0–0.4)	0.06	0.21	**0.035**	0.08	-
Ureaplasma	0 (0–2.1)	0 (0–3.6)	0.02 (0–6.4)	0 (0–2.8)	**0.02**	0.58	**0.004**	0.72	**0.039**

Data are presented as a median fraction of a microorganism in a group with 10 and 90% quantiles. *p*-value, Kruskal–Wallis test. Statistically significant differences are highlighted in bold.

The quantitative estimation of the microorganism representation among all studied groups in HPV(−) patients revealed statistically significant differences in the mean fraction for 4 microorganism genera *Gardnerella*, *Porphyromonas*, *Dialister*, *Campylobacter* (Table 10). Pairwise comparisons showed most differences in the mean microorganism fractions between the ASCUS and NILM groups. On average, the fractions of six microorganism genera (*Gardnerella*, *Porphyromonas*, *Dialister*, *Campylobacter, Prevotella*, *Fusobacterium*) and two family (*Ruminococcaceae, Porphyromonadaceae*) were higher fraction in the ASCUS group than in the NILM group. Other comparisons did not provide any statistically significant results (Supplementary Table S5).

**Table 10:** The quantitative estimation of the microorganism representation in the HPV(−) patients.

Genera	NILM (*n* = 22)	ASCUS (*n* = 11)	LSIL (*n* = 25)	Difference between all groups	ASCUS versus LSIL	ASCUS versus NILM	LSIL versus NILM
				*p*-value			
Prevotella	0 (0–0)	0 (0–21.2)	0 (0–2)	0.1	0.32	**0.036**	0.13
Gardnerella	0 (0–0.6)	6.27 (0–14.9)	0 (0–22)	**0.02**	0.06	**0.004**	0.55
Porphyromonas	0 (0–0)	0 (0–5.79)	0 (0–0)	**0.01**	**0.035**	**0.012**	0.35
Dialister	0 (0–0)	0 (0–7.63)	0 (0–2)	**0.044**	0.54	**0.016**	**0.03**
Campylobacter	0 (0–0)	0 (0–1.01)	0 (0–0)	**0.007**	0.05	**0.003**	0.18
Ruminococcaceae	0 (0–0)	0 (0–2.69)	0 (0–0)	0.075	0.14	**0.04**	0.35
Fusobacterium	0 (0–0)	0 (0–0.7)	0 (0–0.06)	0.15	0.55	**0.04**	0.1
Porphyromonadaceae	0 (0–0)	0 (0–0.08)	0 (0–0)	0.08	0.16	**0.04**	0.35

Data are presented as a median with 10 and 90% quantiles. *p*-value, Kruskal–Wallis test. Statistically significant differences are highlighted in bold.

The microorganisms detected in the ASCUS HPV(+) group (along with the microorganisms listed in Table 11) included *F. vaginae* (with median of 0 and 10% quantile – 90% quantile of 0–8.3) as well as small fractions (90% quantile not exceeding 3%) of *Prevotella* spp., *M. organophilum, Anaerococcus* spp., *F. magna*, *Peptoniphilus* spp., *R. syzygii*, and *R. pickettii*.

In the LSIL HPV(+), *U. urealyticum* and *U. parvum* occurred more frequently and had a higher mean content compared to the other HPV(+) groups. The microorganisms detected in the LSIL group (apart from the microorganisms listed in Table 9): *R. syzygii and R. pickettii* (0%, 0–3.5) as well as small fractions (90% quantile not exceeding 3%) of *F. magna, M. organophilum, Sphingomonas* spp., *Acinetobacter* spp., *Streptococcus* spp., *R. syzygii*, and *R. pickettii*.

In the HSIL HPV(+) groups, the fractions of *F. vaginae*, *G. vaginalis*, *Prevotella* spp. were 0% (0–15.52), 0% (0–23), 0% (0–10.3), respectively. They had the highest occurrence frequencies (Table 7) among all HPV(+) groups. The microbiome of the cervical canal in the HSIL HPV(+) group comprised the following microorganisms (along with the microorganisms listed in Table 9): *Prevotella* spp. (0%, 0–10.3) and *S. sanguinegens* (0%, 0–6.35) as well as small fractions (90% quantile not exceeding 3%) of *Streptococcus* spp., *Anaerococcus* spp., *F. magna, D. micraerophilus, Parvimonas* spp., *Peptoniphilus* spp., *R. syzygii, R. pickettii, U. urealyticum*, *U. parvum, Gemella* spp., *M. massiliensis, and Sphingomonas* spp.

Along with the microorganisms listed in Table 10, the microorganisms detected in the LSIL HPV(−) group included *Bifidobacterium* (0, 0–7.79); *U. urealyticum* and *U. parvum* (0, 0–6.26). They also comprised small fractions (90% quantile not exceeding 3%) of *Sphingomonas* spp., *Peptoniphilus* spp., *F. magna*.

Comparing the quantitative contents of anaerobic microorganisms (*Anaerococcus* spp. - (2.3%, 0–0.1), *D. micraerophilus* (1.8%, 0–1.5), *S. sanguinegens* (6.6%, 0–0.03), *G. vaginalis* (8.3%, 0.2–8.7), *Prevotella* (5.6%, 0–8.7), *Porphyromonas* (1.6%, 0–1.3)) showed that their average percentage was the highest in the ASCUS HPV(−) group compared to the other groups (Table 11).

**Table 11:** Major representatives (species) of the genera detected in this study.

Genus	Major representatives (species)	Abbreviation
*Fannyhessea*	*Fannyhessea vaginae*	*F. vaginae*
*Gardnerella*	*Gardnerella vaginalis*	*G. vaginalis*
*Ralstonia*	*Ralstonia syzygii* *Ralstonia pickettii*	*R. syzygii* *R. pickettii*
*Ureaplasma*	*Ureaplasma urealyticum* *Ureaplasma parvum*	*U. urealyticum* *U. parvum*
*Methylobacterium*	*Methylobacterium organophilum*	*M. organophilum*
*Finegoldia*	*Finegoldia magna*	*F. magna*
*Sneathia*	*Sneathia sanguinegens*	*S. sanguinegens*
*Dialister*	*Dialister micraerophilus*	*D. micraerophilus*
*Megasphaera*	*Megasphaera massiliensis*	*M. massiliensis*

### 3.6 Influence of age and proportion of *Lactobacillus iners* and *Lactobacillus crispatus* on SIL status

The logistic regression based on the age and age of sexual initiation as interacting independent variables and on the SIL status (HSIL/LSIL) as a dependent variable revealed no significant difference for either feature. The HPV status was not included in the model since the HSIL group comprised no HPV(−) patients. The stepwise selection of the best model based on the AIC parameter revealed the statistically significant relationship between the age and an elevated probability of being sorted into the HSIL group rather than LSIL (Table 12; age only model).

**Table 12:** The coefficients (standard error) of the logistic regression models including different type of predictors.

	Age only model	Model with *L. crispatus*	Model with *L. iners*
Age	−0.08^*^ (0.03)	−0.08^*^ (0.035)	−0.1^**^ (0.037)
*Lactobacillus crispatus*	–	0.012^*^ (0.005)	–
*Lactobacillus iners*	–	–	−0.01^*^ (0.006)
Constant	2.78^*^ (1.1)	2.4^*^ (1.12)	3.64 ^**^ (1.23)
Observation	103	103	103
AIC	139.5	136.11	137.13
Dependent variable	SIL status (LSIL or HSIL)		

^*^p < 0.05.; ^**^p < 0.01.

Adding the data on *L. jensenii, L. gasseri* as well as the most represented dominants from CST IV such as *Prevotella*, *Streptococcus*, *Gardnerella*, *Sneathia*, *Fannyhessea* did not exert significant effects on the coefficients for the other features included in the model. The statistical significance remained unaltered, while the absolute value changed only within 2 decimal places. The coefficient for the feature indicating the representation of the taxon based on an absolute value did not exceed 0.05 and was not statistically significant.

Creating the model that includes the values of representation of *L. crispatus, L. iners* did not affect the features previously included in the model as well (Table 12, model with *L. crispatus* and *L. iners*, respectively). The coefficients for these features were statistically significant (*p*-value < 0.05) yet opposite in sign.

In particular, the positive coefficient for *L. crispatus* shows that an elevated percentage leads to an increase of the probability of detecting LSIL rather than HSIL. At the same time, elevated content of *L. iners* results in a higher probability of detecting HSIL rather than LSIL given the equal age of a patient.

### 3.7 The predominant *Lactobacillus* species patients with normocenosis, moderate and severe dysbiosis (anaerobic, aerobic, and mixed)

**Figure 5:**
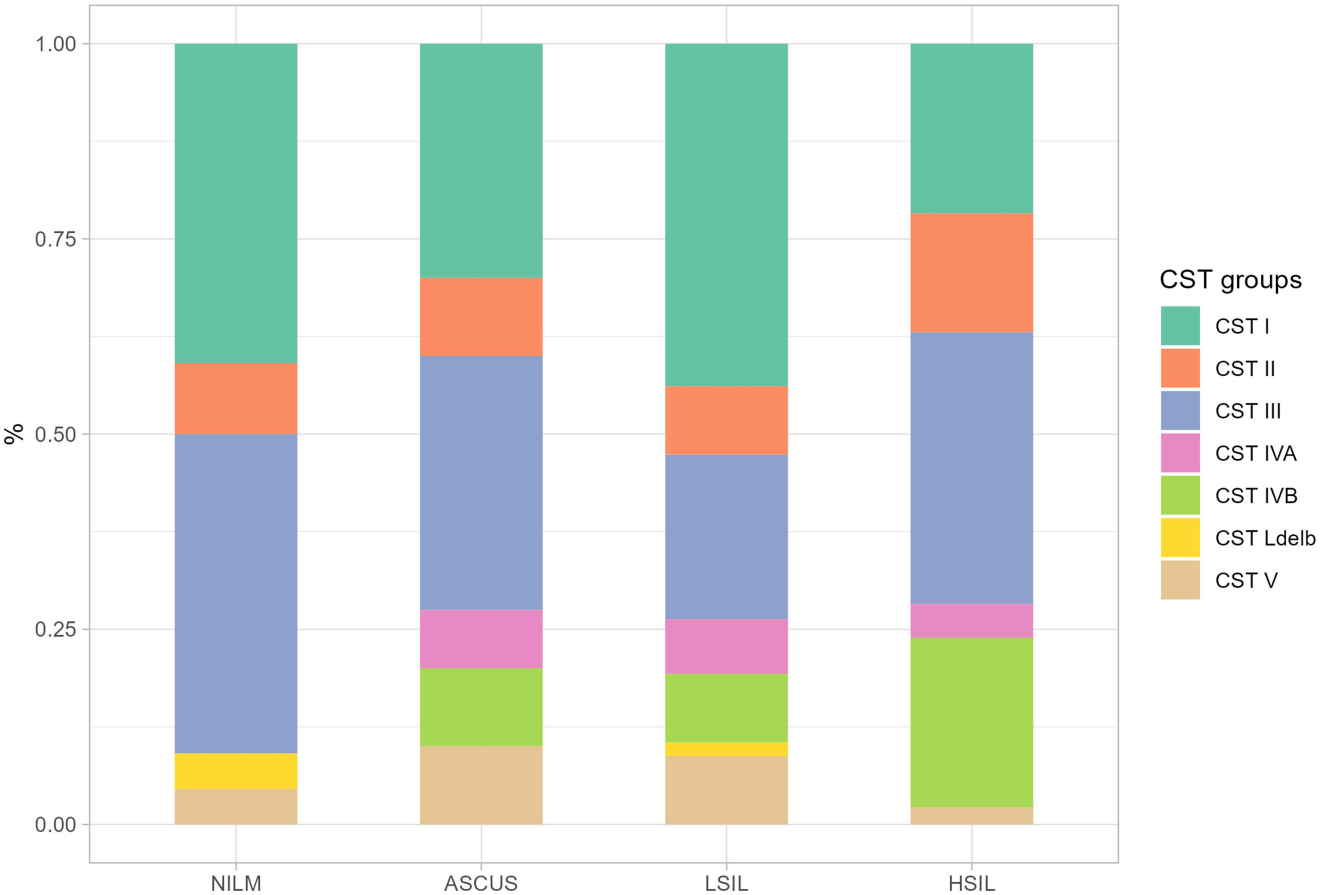
The ratio of CST fraction in the groups under scrutiny. The plot shows the proportion of samples from different. All CST groups was determined by predominant *Lactobacillus* (CST I – CST III, CST V, CST Ldelb) or other non-*Lactobacillus* (CST IVA, CST IVB) genus.

In this study, we detected all previously isolated CST I-V including IV-A and IV-B (Figure 5). However, in two patients (one was from the NILM group, the other one - from the LSIL group), *L. delbrueckii* prevailed with the contents of 90 and 74%, respectively. CST was termed СST Ldelb in these patients.

Among all groups under investigation, the predominant microorganisms were CST I and II, with *L. crispatus* and *L. iners* prevailing in the cervical canal. The highest fraction of the CST IV group which correlates with pathologic microflora state was observed in the HSIL group (Figure 5).

In the HSIL group, the most severe dysbiosis was found in 24% of patients being the most common dysbiosis among all studied groups. In the patients with the established normocenosis, *L. delbrueckii* was absent in all the groups (ASCUS, LSIL, HSIL).

**Figure 6:**
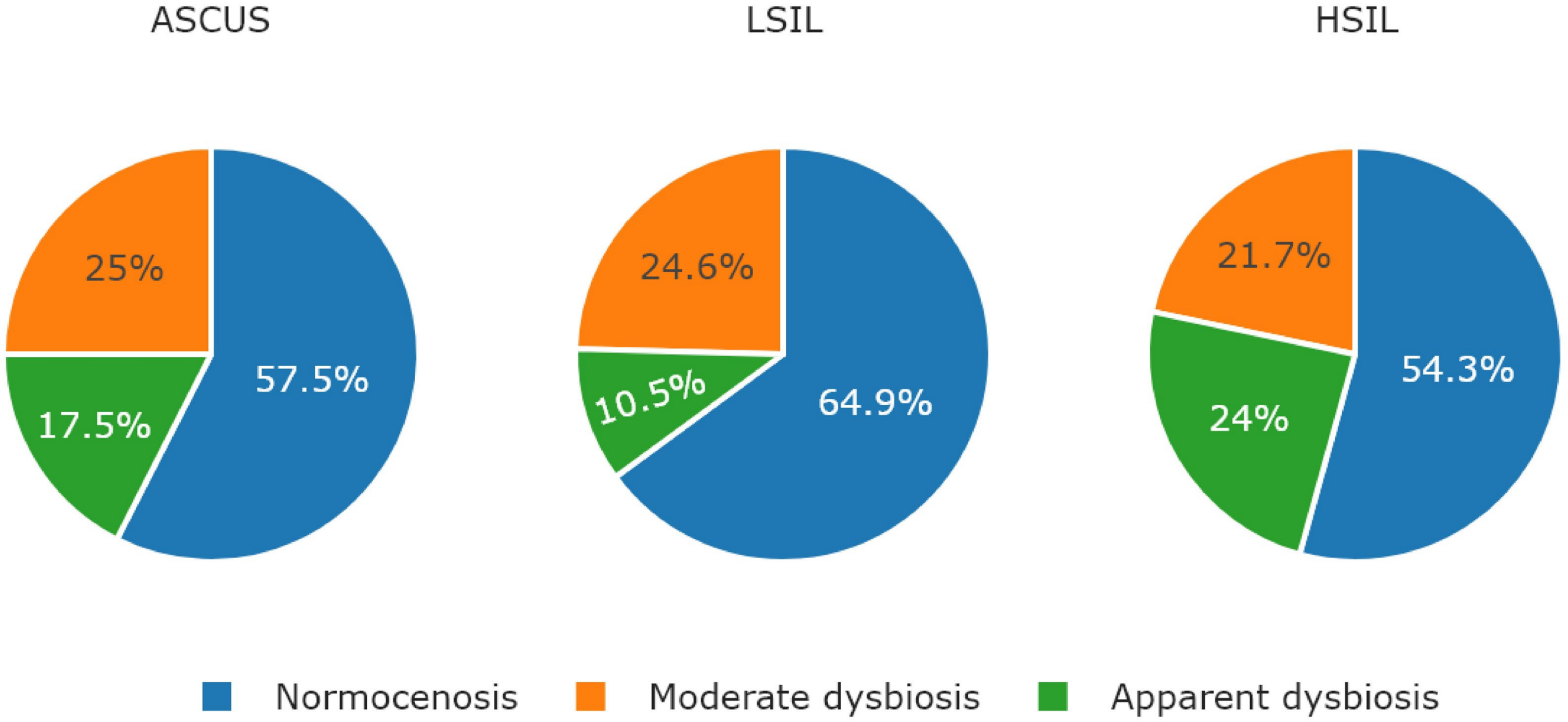
The ratio of microbiological cenosises were observed in the studied groups. The type of cenosis was determined by the percentage of *Lactobacillus* genus in the sample. Normocenosis – more than or equal 80%; moderate dysbiosis – from 20 to 80%; apparent dysbiosis – <20%.

**Figure 7:**
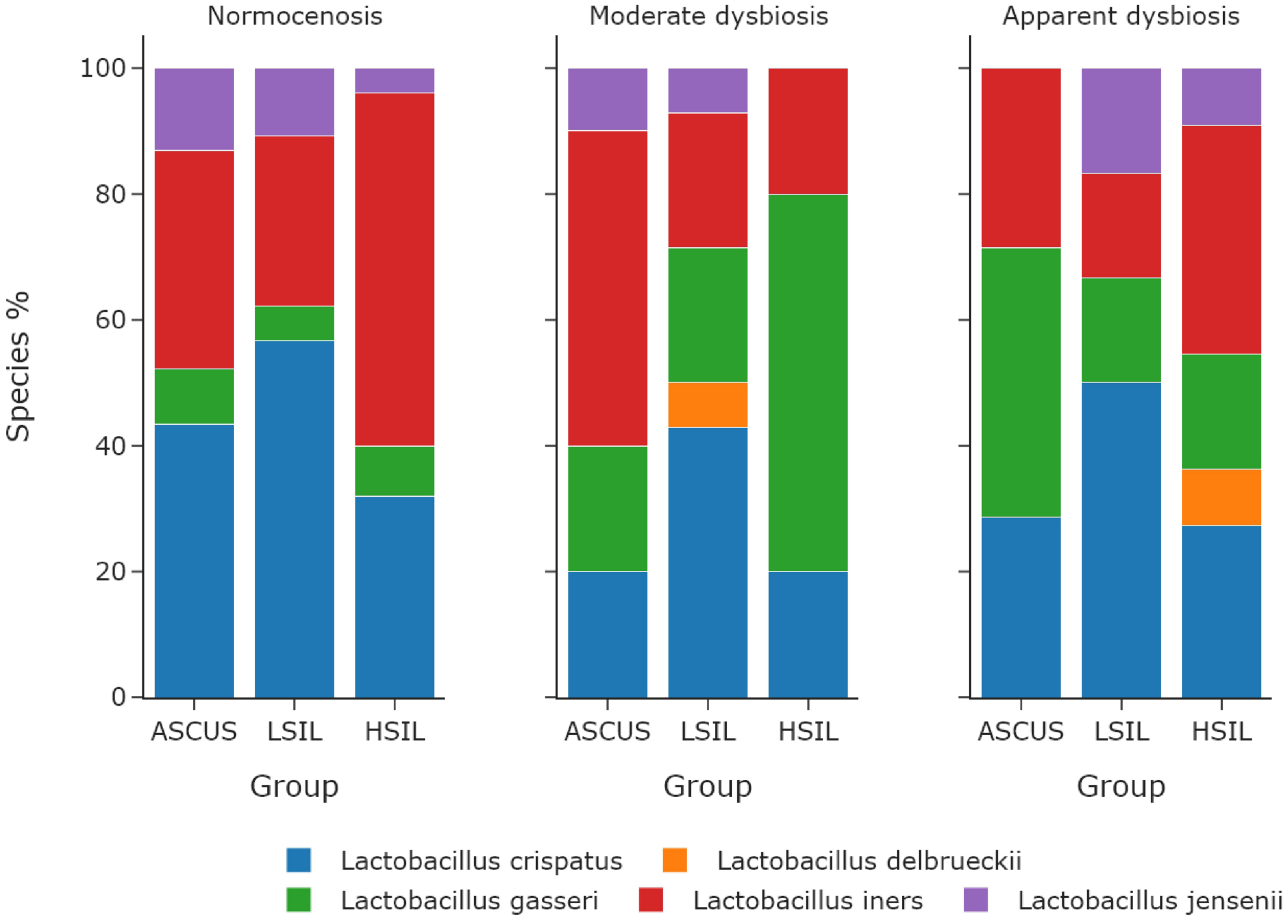
The dependence of the fraction of predominant *Lactobacillus* species on microbiome cenosises. The type of cenosis was determined by the percentage of *Lactobacillus* genus in the sample. Normocenosis – more than or equal 80%; moderate dysbiosis – from 20 to 80%; apparent dysbiosis – <20%.

The microorganisms most frequently prevailing in the NILM, ASCUS, LSIL, HSIL groups were *L. crispatus*, *L. iners*, and *L. gasseri* (Figures 6, 7; Table 13). The highest *L. gasseri* content was observed in the HSIL patients. The *L. jensenii* fraction was relatively small. The fraction of the samples containing *L. crispatus* was the smallest in the HSIL group. Furthermore, in normocenosis, *L. gasseri* content was the smallest in all groups.

**Table 13:** The dependence of predominant *Lactobacillus* species on cenosis in a sample.

Group	State	*n* (% of total amount patients)	Predominant *Lactobacillus* species	*n* of the predominant species (% of total amount cenosis)
NILM (*n* = 22)	Normocenosis	22 (100)	*L. iners/L. crispatus*	9 (40.9)/9 (40.9)
ASCUS (*n* = 40)	Normocenosis	23 (57.5)	*L. crispatus*	10 (43.5)
	Moderate dysbiosis	10 (25)	*L. iners*	5 (50)
	Apparent dysbiosis	7 (17.5)	*L. gasseri*	3 (42.9)
LSIL (*n* = 57)	Normocenosis	37 (65)	*L. crispatus*	21 (56.8)
	Moderate dysbiosis	14 (24.6)	*L. crispatus*	6 (42.9)
	Apparent dysbiosis	6 (10.5)	*L. crispatus*	3 (50)
HSIL (*n* = 46)	Normocenosis	25 (54.3)	*L. iners*	14 (56)
	Moderate dysbiosis	10 (21.7)	*L. gasseri*	6 (60)
	Apparent dysbiosis	11 (24)	*L. iners*	4 (36.4)

In each microbiota state (normocenosis, moderate dysbiosis, pronounced dysbiosis), HSIL samples exhibit a certain decrease of the representation of *L. crispatus* with the predominance of other species (Table 13; Supplementary Table S6).

## 4 Discussion

Among women with cervical dysfunction confirmed by cytological and/or histological examination, cervical HPV colonization was observed in 100, 72.5, and 56.1% for HSIL, ASCUS, and LSIL, respectively. The most common HPV type was HPV16 which occurred twice as often in the patients with severe cervical dysfunction (HSIL, 58.8%) compared to LSIL (28%) and ASCUS (25%) patients. Alpha and beta diversity did not show any statistically significant difference between studied group. We suggest that it may be due to lack of influence of HPV to cervical canal microbiota. Nevertheless, some CST (for example CST IV or CST III) could be more vulnerable to entry and spread of HPV. In healthy women of a reproductive age, equally often prevailing types of Lactobacillus are *L. crispatus* and *L. iners*. For example, *L. crispatus* turned out to be the predominant *Lactobacillus* species in women with normocenosis for the ASCUS and LSIL groups, while *L. iners* prevailed in the HSIL group. There are contradictions in the works of other authors concerning *L. iners*: some studies show that it accompanies HPV infection and SIL (Wang et al., 2019; Sasivimolrattana et al., 2022; Xu et al., 2022), whereas other studies demonstrate no association of the kind (McKee et al., 2020; Gomez Cherey et al., 2023; Ivanov et al., 2023). *L. crispatus* is considered to protect from SIL progressing in HPV (Cheng et al., 2020), since it produces a lot of the D-isomer of lactic acid which increases the viscosity of the vaginal secrete and elevates its ability to sequester virions, in contrast to *L. iners* which produces only the L-isomer (Kalia et al., 2020; Nicolò et al., 2023). Moreover, *L. iners* produces inerolysin that creates pores in the vaginal epithelium potentially promoting HPV infection (Xu et al., 2022; Sharifian et al., 2023). This theory agrees to our results. All taxons with statistically significant difference are usually found in cervical microbiota, but they have higher occurrence and ratio in HSIL group compared with NILM and LSIL groups. The logistic regression allowed for individually evaluating the impact of the age and the role of certain microorganisms in HSIL and LSIL development. The older was the patient as well as the higher was *L. iners* fraction in the sample from the cervical canal, the higher was the probability of detecting HSIL rather than LSIL. Conversely, the younger age and high *L. crispatus* content increased the chance of being sorted into the LSIL group. In our work, the ASCUS patients with moderate dysbiosis more often contained *L. iners,* whereas LSIL patients contained *L. crispatus,* and HSIL patients had *L. gasseri.* Severe dysbiosis correlated with *L. gasseri*, *L. crispatus,* and with both *L. crispatus and L. iners* in the ASCUS, LSIL, and HSIL groups, respectively. *L. gasseri* was the least common *Lactobacillus* in the women of reproductive age. *L. delbrueckii* was the predominant *Lactobacillus* being present in 1.8% of the enrolled women, although it did not belong to any type of vaginal communities. In the ASCUS group, LSIL, and HSIL dysbiosis were observed in 42.5, 35, and 45.6% of patients, respectively. Anaerobic dysbiosis or bacterial vaginosis was the most prevalent in all groups. In bacterial vaginosis, the following anaerobic microorganisms prevailed: *G. vaginalis, F. vaginae, D. micraerophilus, S. sanguinegens, Anaerococcus* spp., *M. massiliensis, Prevotella* spp., *F. magna, Peptoniphilus* spp., *Porphyromonas* spp., *and Parvimonas* spp. Aerobic vaginitis was more rare, and in all cases under scrutiny, the predominant microorganism to cause it was *Streptococcus* spp. The bacteria associated with bacterial vaginitis produce carcinogenic nitrosamines, which trigger the release of cytokines such as interleukine-1b involved in SIL development. During HPV infection, the cytokines weaken the immune system, while carcinogenic nitrosamines facilitate DNA damage (Muzny et al., 2020; Tidbury et al., 2021). The predominance of certain groups of microorganisms such as *Sneathia, Prevotella, Megasphaera, Dialister, Gardnerella, Streptococcus*, and *Fannyhessea* is associated with SIL development (Mitra et al., 2020; Dong et al., 2022) which is in line with our data. However, it is believed that the predominance of anaerobic microorganisms is associated with LSIL (Lin et al., 2021), whereas aerobic vaginitis is associated with HSIL (Plisko et al., 2021). Colonization of the cervical canal and vagina by *U. urealyticum* and *U. parvum* lead to SIL development (Condic et al., 2023) which is supported by our results, since *Ureaplasma* spp. prevailed both qualitatively and quantitatively in the LSIL HPV(+) patients. Other works suggest that microbial diversity is elevated upon HPV infection (Chen et al., 2020; Usyk et al., 2020) which, however, was not observed in this work (Figure 7).

The qualitative and quantitative comparisons of microbiomes between the groups allowed us to detect statistically significant differences for several genera: *Prevotella, Gardnerella, Porphyromonas, Dialister*, *Staphylococcus, Ureaplasma*. Estimating the possible relationship of various *Lactobacillus* species with dysbiotic impairments of the microbiome and HPV-associated cervical diseases did not reveal any statistical significant difference in the *Lactobacillus* species composition and its relationship with HPV-associated cervical disorders. Of note, that *L. iners, L. gasseri, L. crispatus* were often detected upon dysbiosis, whereas *L. gasseri* was the least common in all groups. Evaluating dysbiotic impairments of the microbiome in HPV(+) patients based on 16S sequencing demonstrated that dysbiosis was most common in the patients with ASCUS (42.5%), LSIL (35%), and HSIL (45.6%). Bacterial vaginosis was associated with *G. vaginalis, F. vaginae, D. micraerophilus, S. sanguinegens, Anaerococcus* spp., *M. massiliensis, Prevotella* spp., *F. magna, Peptoniphilus* spp., *Porphyromonas* spp., and *Parvimonas* spp., while aerobic vaginitis was related to *Streptococcus* spp. Detecting HPV along with the impaired microbiome, such as bacterial vaginosis and aerobic vaginitis, may allow for identifying the groups of women with the high risk of SIL development and progression.

## Data availability statement

The data presented in the study are deposited in the NCBI BioProject repository, accession number PRJNA1041987.

## Ethics statement

The studies involving humans were approved by Local Ethics Committee at the FSBI “National Medical Research Center for Obstetrics, Gynecology and Perinatology Named After Academician V.I.Kulakov” Ministry of Health of the Russian Federation (protocol no. 3 of 26 March 2020). The studies were conducted in accordance with the local legislation and institutional requirements. The participants provided their written informed consent to participate in this study.

## Author contributions

AP: Conceptualization, Formal analysis, Investigation, Methodology, Visualization, Writing – original draft. VC: Data curation, Formal analysis, Investigation, Methodology, Software, Validation, Visualization, Writing – original draft, Writing – review & editing. AK: Conceptualization, Investigation, Methodology, Software, Writing – original draft. AAn: Conceptualization, Investigation, Methodology, Writing – original draft. ZR: Conceptualization, Data curation, Formal analysis, Software, Visualization, Writing – original draft. AAs: Conceptualization, Investigation, Writing – original draft. DK: Funding acquisition, Project administration, Resources, Supervision, Writing – review & editing. DR: Funding acquisition, Project administration, Resources, Supervision, Writing – review & editing. GB: Conceptualization, Project administration, Resources, Supervision, Validation, Writing – original draft, Writing – review & editing.
